# Organ-specific, integrated omics data-based study on the metabolic pathways of the medicinal plant *Bletilla striata* (Orchidaceae)

**DOI:** 10.1186/s12870-021-03288-9

**Published:** 2021-11-01

**Authors:** Xiaoxia Ma, Kehua Tang, Zhonghai Tang, Aiwen Dong, Yijun Meng, Pu Wang

**Affiliations:** 1 College of Pharmaceutical Science, Zhejiang University of Technology, Hangzhou, 310014 China; 2 School of Pharmacy, Hangzhou Normal University, Hangzhou, 311121 China; 3 Key Laboratory of Hunan Forest Products and Chemical Industry Engineering, Jishou University, Zhangjiajie, 427000 China; 4 College of Food Science and Technology, Hunan Agricultural University, Changsha, 410128 China; 5 College of Life and Environmental Sciences, Hangzhou Normal University, Hangzhou, 311121 China

**Keywords:** Organ-specific, Transcriptome assembly, Metabolic pathways, Gene expression, Long-range transportation, *Bletilla striata*

## Abstract

**Background:**

*Bletilla striata* is one of the important species belonging to the *Bletilla* genus of Orchidaceae. Since its extracts have an astringent effect on human tissues, *B. striata* is widely used for hemostasis and healing. Recently, some other beneficial effects have also been uncovered, such as antioxidation, antiinflammation, antifibrotic, and immunomodulatory activities. As a key step towards a thorough understanding on the medicinal ingredient production in *B. striata*, deciphering the regulatory codes of the metabolic pathways becomes a major task.

**Results:**

In this study, three organs (roots, tubers and leaves) of *B. striata* were analyzed by integrating transcriptome sequencing and untargeted metabolic profiling data. Five different metabolic pathways, involved in polysaccharide, sterol, flavonoid, terpenoid and alkaloid biosynthesis, were investigated respectively. For each pathway, the expression patterns of the enzyme-coding genes and the accumulation levels of the metabolic intermediates were presented in an organ-specific way. Furthermore, the relationships between enzyme activities and the levels of the related metabolites were partially inferred. Within the biosynthetic pathways of polysaccharides and flavonoids, long-range phytochemical transportation was proposed for certain metabolic intermediates and/or the enzymes.

**Conclusions:**

The data presented by this work could strengthen the molecular basis for further studies on breeding and medicinal uses of *B. striata*.

**Supplementary Information:**

The online version contains supplementary material available at 10.1186/s12870-021-03288-9.

## Background

Orchidaceae is a large plant group of great research value, not only due to its ornamental feature, but also thanks to its diversified phytochemicals with pharmacological activities [1]. As one of the orchid species, *Bletilla striata* (Thunb.) Reichb. f. (Orchidaceae) is a perennial herbal bulbous plant belonging to the *Bletilla* genus. Although treated as an ornamental plant in most of the Western countries, *B. striata* is widely used in the traditional medicine formulations in many Asian countries. Especially in China, the dried tubers of *B. striata*, recorded as “Bai ji” in Chinese pharmacopoeia (2010), has been used as a traditional Chinese medicine (TCM) for thousands of years. In the modern times, many beneficial health effects of *B. striata*, such as healing, hemostasis, antioxidation, antiinflammation, antifibrotic and immunomodulatory activities, have been uncovered by the recent phytochemical and pharmacological studies [2, 3]. Recently, China Food and Drug Administration has approved four patent medicines including “Bai ji” Pill, “Bai ji” Capsule, “Bai ji” Syrup, and “Bai ji” Granule, all of which contain *B. striata* as the unique medicinal ingredient [2]. Complicatedly, the medicinal effects of *B. striata* may be contributed by its diversified functional ingredients. To date, hundreds of chemical compounds have been identified from *B. striata*, which were classified into several categories, such as glucosides, bibenzyls, phenanthrenes, quinones, biphenanthrenes, dihydrophenanthrenes, anthocyanins, steroids, triterpenoids and phenolic acids [4]. *B. striata* is also a valuable resource for medical, cosmetic and some other industries. For example, it was widely used for the production of embolizing agent, mucosa-protective agent, cosmetics, mucilage and some other biomaterials [2, 4], which led to the increasing market demand for *B. striata*. Excessive exploitation along with the low reproduction rate of *B. striata* has made the wild resources scarce. Several years ago, *B. striata* was listed as one of the wild preferential conservation medicinal plants [5].

The lack of well-annotated genomic information of the non-model plant *B. striata* has been partially solved by the other ever-improving omics research strategies. Omics data-based analysis of metabolic pathways in medicinal plants has become one of the hot topics on variety selection and breeding at the molecular level. To date, several omics data-based studies have been performed for *B. striata* [6–11]. Notably, most of these studies were performed through transcriptome sequencing. For example, Niu and his colleagues (2020) analyzed the enzymes involved in polysaccharide biosynthesis based on the *de novo* assembled transcriptome of *B. striata*. In recent years, the strategy by integrating transcriptomic data with metabolomic data was employed for the studies on several medicinal plants, such as *Cistanche deserticola* [7] and *Ginkgo biloba* [12]. However, no such comprehensive study has been reported for *B. striata*.

In this study, an omics data-based analysis of the metabolic pathways was performed for the two-year-old seedlings of *B. striata*. Considering the fact that the dried tubers of *B. striata* was used as the major medicinal part for TCM, three organs including roots, tubers and leaves were sampled for transcriptome sequencing and metabolome profiling. Five different metabolic pathways, related to polysaccharide, sterol, flavonoid, terpenoid and alkaloid biosynthesis respectively, were investigated in an organ-specific manner by integrated analysis of the transcriptome and the metabolome data. For each pathway, the relationship between the enzyme activities and the metabolite levels were partially inferred. Moreover, a hypothesis of long-range phytochemical transportation was proposed for the metabolic intermediates and/or the enzymes belonging to the polysaccharide and the flavonoid biosynthetic pathways. Summarily, our work presented here could strengthen the molecular basis for further studies on breeding and medicinal uses of *B. striata*.

## Results

### Untargeted metabolic profiling and transcriptome sequencing

**Fig. 1:**
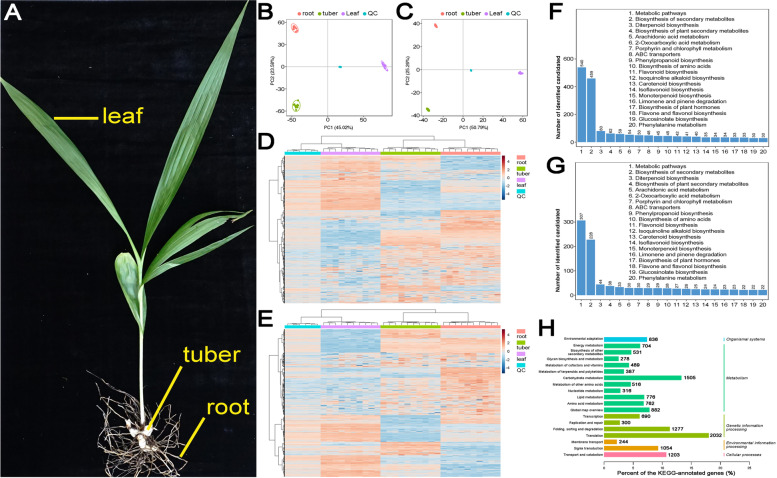
Organ-specific metabolic profiles of *Bletilla striata*. **A** Leaves, tubers and roots of the two-year old seedlings of *B. striata* were collected for untargeted metabolic profiling and transcriptome sequencing. **B** Principal component analysis (PCA) indicates that the metabolic profile obtained under the positive ion mode has an organ-specific feature. **C** PCA indicates that the metabolic profile obtained under the negative ion mode also has an organ-specific feature. **D** A heatmap showing the levels of the metabolites identified from the three organs of *B. striata* under the positive ion detection mode of ultra-performance liquid chromatography—tandem mass spectrometry (UPLC-MS/MS). **E** A heatmap showing the levels of the metabolites identified from the three organs of *B. striata* under the negative ion detection mode of UPLC-MS/MS. Based on metabolite quantification, the heatmaps were drawn at a log2 scale from -4 to +4. **F** Histogram showing the top 20 KEGG pathways, to which the MS1-level metabolites identified under the positive ion mode were annotated. **G** Histogram showing the top 20 KEGG pathways, to which the MS1-level metabolites identified under the negative ion mode were annotated. **H** KEGG-based annotations of the transcriptome assembly

In order to obtain an organ-specific metabolic profile, three different organs, including roots, tubers and leaves, were collected from the two-year-old seedlings of *B. striata* (Fig. 1A). For each organ, ten biological replicates were included for untargeted metabolic profiling by UPLC-MS/MS analysis. As a result, a total of 7,736 and 3,041 features were identified in positive and negative ion modes. Among these features, 3,388 (43.80%) and 1,247 (41.00%) were regarded as the candidate metabolites MS1-level, and were successfully annotated by the KEGG (Kyoto Encyclopedia of Genes and Genomes) database [13]. At first glance, the result of PCA (principal component analysis) showed that the global features of the total extracts of the three organs were extremely distinguishable under both positive and negative ion detection modes. Moreover, for each organ, the ten replicates were highly consistent (Fig. 1B and C). Signal intensity-based clustering analysis of the metabolomes from different organs indicated that the two underground organs (i.e. roots and tubers) were more similar to each other when compared to leaves (Fig. 1D and E). The metabolites identified at the MS1-level were further analyzed based on the KEGG annotations. The result showed that among the top 20 KEGG pathways, to which the MS1 metabolites were annotated, a large portion were involved in secondary metabolism, such as monoterpenoid and diterpenoid biosynthesis, flavonoid and isoflavonoid biosynthesis, flavone and flavonol biosynthesis, anthocyanin biosynthesis, pentose and glucuronate interconversion, galactose metabolism, isoquinoline alkaloid biosynthesis, and biosynthesis of plant hormones (Fig. 1F and G).

Transcriptome sequencing was also performed for the three organs with three biological replicates. A total of 69.47 GB valid sequencing data was combinatorially used to assemble the transcriptome of *B. striata*. As a result, 111,628 transcripts assigned to 42,974 genes were obtained. Based on the KEGG database, a total of 11,282 genes were annotated (Additional file 1). Notably, more than 50% of these KEGG-annotated genes participate in diverse metabolic pathways, including glycan biosynthesis and metabolism, terpenoid and polyketide metabolism, lipid metabolism, and biosynthesis of other secondary metabolites (Fig. 1H).

Taken together, the above results demonstrate that a significant portion of the metabolites and the genes identified from *B. striata* are involved in the metabolic pathways covering glycan, phytohormone, flavonoid, terpenoid, and alkaloid biosynthesis. In this regard, the above five synthetic pathways were further investigated as follows.

### BSP biosynthetic pathway

*Bletilla striata* polysaccharides (BSPs) are usually modified or cross-linked with other chemical substances, in order to be used as the biomaterials for wound healing and drug delivery [14, 15]. BSPs are largely composited by the monosaccharides mannose and glucose, thus could be regarded as the glucomannan polymers [16]. Although the biosynthetic pathway of BSPs have been recently studied by using transcriptome sequencing data [10], we would like to uncover the detailed regulatory mechanism by integrating metabolic data through an organ-specific way.

**Fig. 2:**
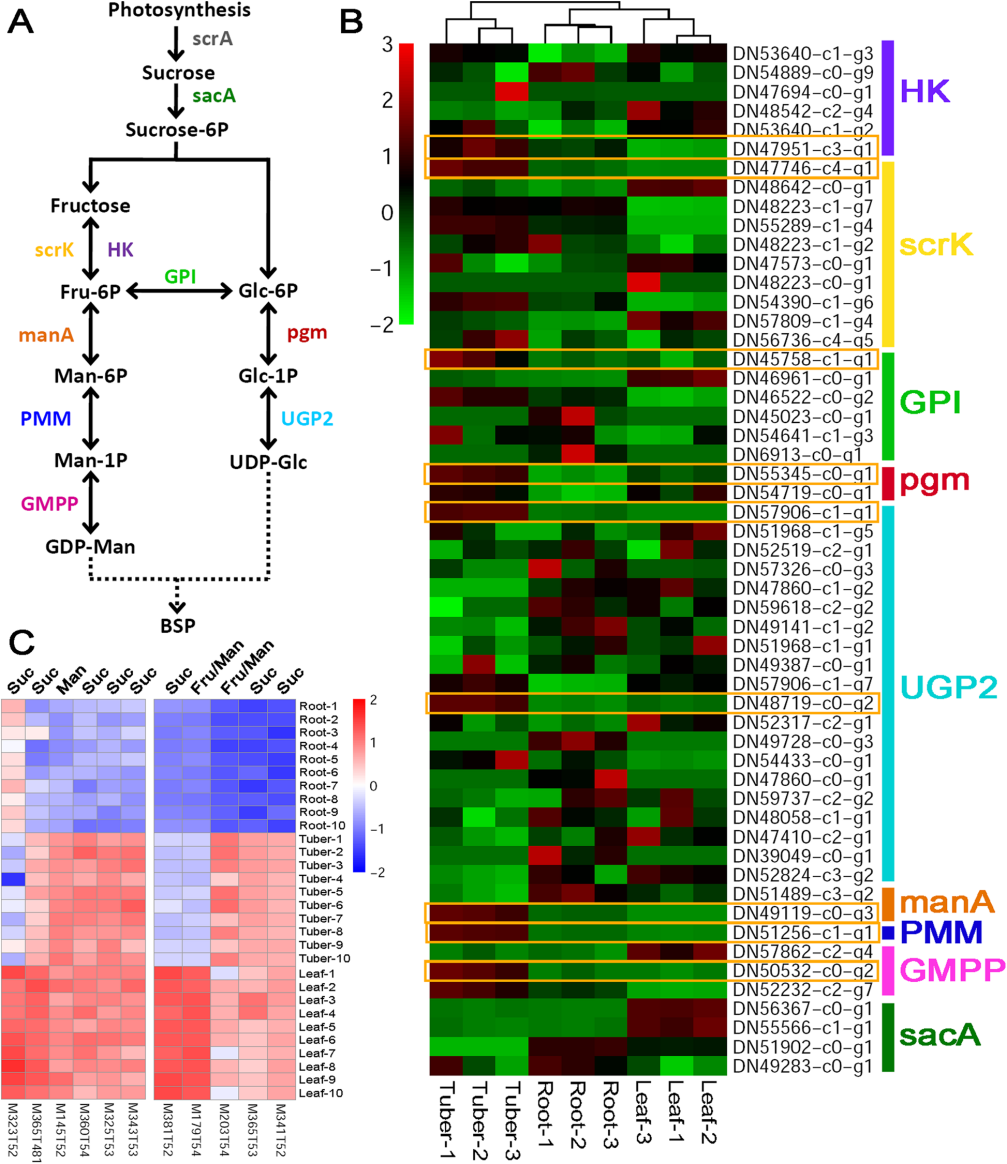
Organ-specific analysis of the levels of the enzyme-coding genes and the metabolites within the biosynthetic pathway of *Bletilla striata* polysaccharide (BSP). **A** The previously reported biosynthetic pathway of BSP [10] was employed for this analysis. Based on the transcriptome assembly annotated by the KEGG database, nine enzymes could find their coding genes (colored within the pathway) based on the EC numbers. scrA: phosphotransferase system, [EC:2.7.1.211]; sacA: beta-fructofuranosidase, [EC:3.2.1.26]; HK: hexokinase, [EC:2.7.1.1]; scrK: fructokinase, [EC:2.7.1.4]; GPI: glucose-6-phosphate isomerase, [EC:5.3.1.9]; pgm: phosphoglucomutase, [EC:5.4.2.2]; UGP2: uridine-diphosphate glucose pyrophosphorylase, [EC:2.7.7.9]; manA: mannose-6-phosphate isomerase, [EC:5.3.1.8]; PMM: phosphomannomutase, [EC:5.4.2.8]; GMPP: mannose-1-phosphate guanylyltransferase, [EC:2.7.7.13]. **B** A heatmap showing the expression patterns of the enzyme-coding genes in roots, tubers and leaves of *B. striata*. In most cases, there are two or more coding genes for each enzyme. After *z*-score normalization, the heatmap was drawn at a log2 scale from -2 to +3. **C** A heatmap showing the levels of the metabolites in three organs. Based on metabolite quantification, the heatmaps were drawn at a log2 scale from -2 to +2. Suc: sucrose, C00089; Fru: fructose, C01496, C02336; Man: mannose, C00159

Referring to the pathway reported by Niu and his colleagues (2020), there are ten enzymes critical for BSP biosynthesis. In our study, a total of nine enzymes (colored enzymes, except for the grey one in Fig. 2A) could find their coding genes from the transcriptome assembly. In many cases, two or more coding genes were assigned to a specific enzyme, indicating their functional redundancy or complementarity (Fig. 2B). Expression data-based analysis showed that except for sacA (beta-fructofuranosidase, [EC:3.2.1.26]), at least one coding gene of each enzyme was found to be highly expressed in tubers (marked by the yellow boxes in Fig. 2B, and Additional file 2), when compared to the other two organs. From this point of view, a highly active catalytic system may exist in the tubers for midstream and downstream production of BSPs. In fact, the tubers are the traditionally used medicinal part enriched with BSPs [3].

To further investigate the relationship between the levels of the metabolites and the activities of the enzyme-coding genes within the BSP synthetic pathway, the untargeted metabolic profiling data was analyzed. A total of 11 metabolites were detected at the MS1 level, and were annotated as sucrose (KEGG ID: C00089), fructose (C01496 and C02336) and mannose (C00159). Interestingly, the metabolites were most abundant in leaves, and followed by tubers. The levels of sucrose, fructose and mannose are especially low in roots (Fig. 2C). The enrichment of the metabolites belonging to the BSP pathway in the leaf organ might be attributed to its capacity of photosynthesis. Possibly, the leaves serve as a source organ for the tubers that could be regarded as a sink organ with highly activated enzymatic system for the downstream production of BSPs. Detailed mechanism will be discussed below.

### Sterol biosynthetic pathway

Phytosterols are the precursors for the synthesis of brassinosteroids (BRs) which were known as one of the essential hormones regulating plant development and stress response [17]. In *Arabidopsis thaliana*, the *BREVIS RADIX* (*BRX*) gene, involved in root growth regulation, was reported to activate the genes participating in BR synthesis [18]. On the other hand, both roots and tubers are usually used as the explants for manual propagation of *B. striata* [5]. Thus, it will be interesting to investigate the sterol biosynthetic pathway in *B. striata*.

**Fig. 3:**
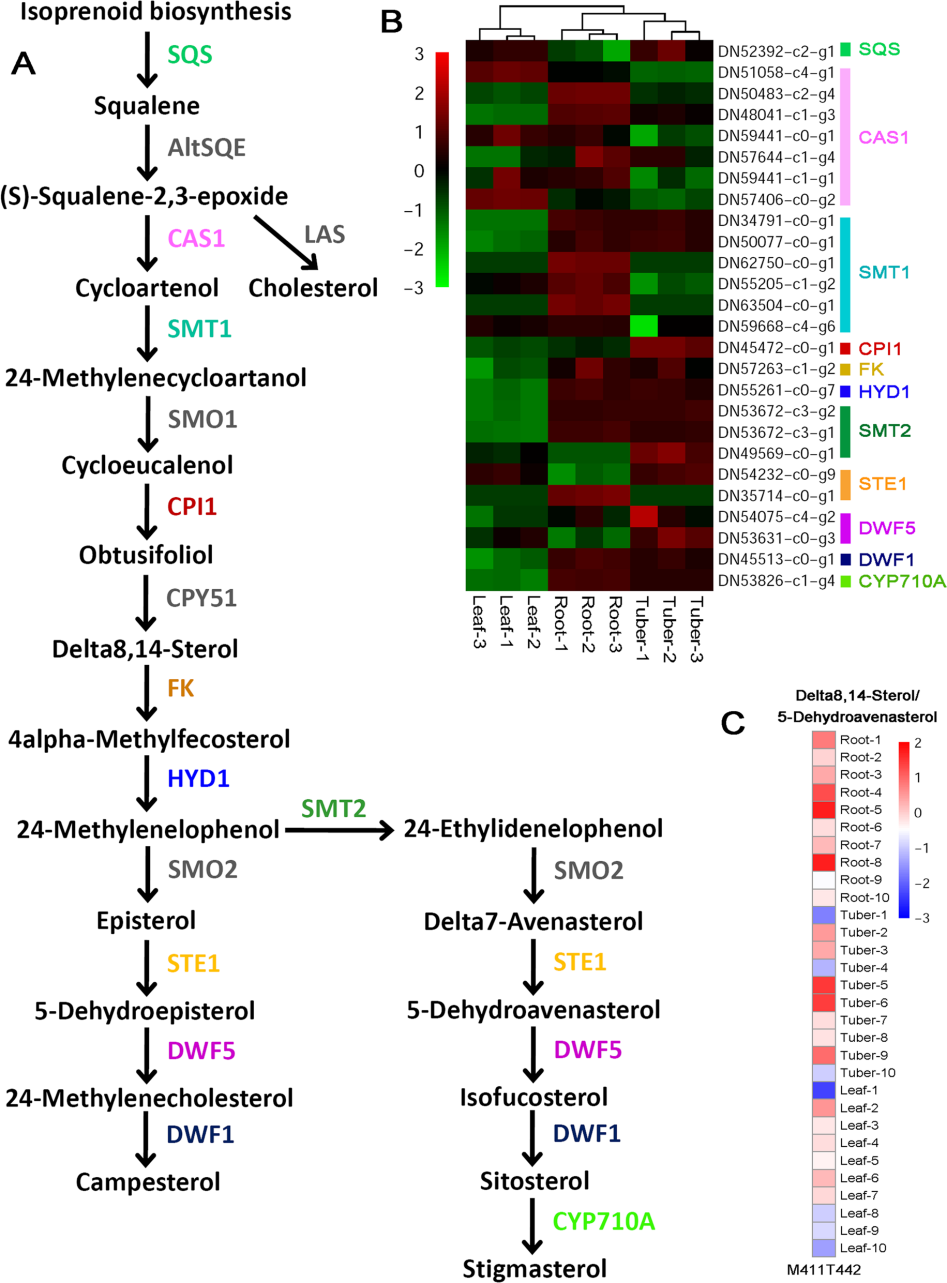
Organ-specific analysis of the levels of the enzyme-coding genes and the metabolites within the sterol biosynthetic pathway of *Bletilla striata*. **A** The previously reported biosynthetic pathway of sterols [17]. Based on the transcriptome assembly annotated by the KEGG database, 11 enzymes could find their coding genes (colored within the pathway) based on the EC numbers. SQS: squalene synthase, [EC:2.5.1.21]; AltSQE: alternative squalene epoxidase, [EC:1.14.19.-]; LAS: lanosterol synthase, [EC:5.4.99.7]; CAS1: cycloartenol synthase, [EC:5.4.99.8]; SMT1: sterol 24-C-methyltransferase, [EC:2.1.1.41]; SMO1: plant 4,4-dimethylsterol C-4alpha-methyl-monooxygenase, [EC:1.14.18.10]; CPI1: cycloeucalenol cycloisomerase, [EC:5.5.1.9]; CPY51: sterol 14alpha-demethylase, [EC:1.14.14.154], [EC:1.14.15.36]; FK: Delta14-sterol reductase, [EC:1.3.1.70]; HYD1: cholestenol Delta-isomerase, [EC:5.3.3.5]; SMT2: 24-methylenesterol C-methyltransferase, [EC:2.1.1.143]; SMO2: plant 4alpha-monomethylsterol monooxygenase, [EC:1.14.18.11]; STE1: Delta7-sterol 5-desaturase, [EC:1.14.19.20]; DWF5: 7-dehydrocholesterol reductase, [EC:1.3.1.21]; DWF1: Delta24-sterol reductase, [EC:1.3.1.72], [EC:1.3.1.-]; CYP710A: sterol 22-desaturase, [EC:1.14.19.41]. **B** A heatmap showing the expression patterns of the enzyme-coding genes in roots, tubers and leaves of *B. striata*. For some of the enzymes, there are two or more coding genes. After *z*-score normalization, the heatmap was drawn at a log2 scale from -3 to +3. **C** A heatmap showing the levels of the metabolite in three organs. Based on metabolite quantification, the heatmaps were drawn at a log2 scale from -3 to +2. Delta8,14-Sterol: C11508; 5-Dehydroavenasterol: C15783

Based on the reference pathway proposed for the model plants *Arabidopsis* and rice [17], a total of 11 enzymes within the sterol synthetic pathway (colored enzymes, except for the grey ones in Fig. 3A) could find their coding sequences from the transcriptome assembly. Transcriptome sequencing data-based expression analysis showed that most of the enzyme-coding genes were highly activated in roots and/or tubers, when compared to leaves (Fig. 3B and Additional file 2). Although only one metabolite (M411T442, annotated as 5-dehydroavenasterol/delta 8,14-sterol) was identified by the untargeted metabolic profiling, it was observed that the levels of this metabolite in roots and tubers were much higher than that in leaves (Fig. 3C). Notably, 5-dehydroavenasterol is one of the precursors proximal to the end product stigmasterol within the sterol biosynthetic pathway. In this regard, the biosynthesis of sterols is much more active in roots and tubers of *B. striata*, which could be essential for the growth regulation of the two organs.

### Flavonoid biosynthetic pathway

Flavonoids, the most abundant polyphenols in plants, are one important type of the plant secondary metabolites. Multiple pharmacological effects of the plant flavonoids have been uncovered, such as antioxidation [19], antiinflammation [20] and anticancer [21]. The biosynthetic pathway of the flavonoids has been studied in the seeds of *Arabidopsis* [22] and the leaves of *Ginkgo biloba* [12]. However, the scene of flavonoid biosynthesis is poorly understood in *B. striata*.

**Fig. 4:**
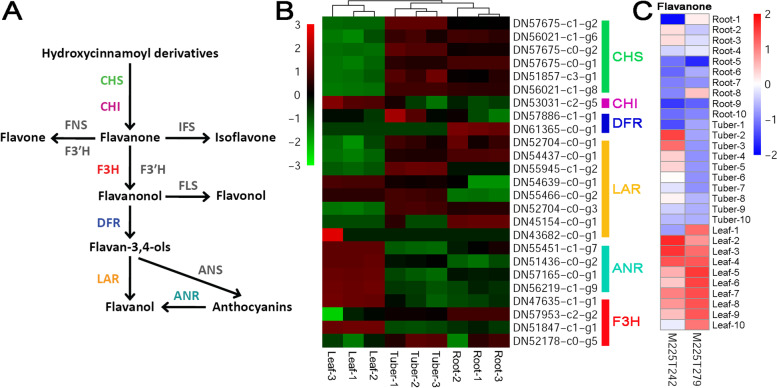
Organ-specific analysis of the levels of the enzyme-coding genes and the metabolites within the flavonoid biosynthetic pathway of *Bletilla striata*. **A** The previously reported biosynthetic pathway of flavonoids [12]. Based on the transcriptome assembly annotated by the KEGG database, six enzymes could find their coding genes (colored within the pathway) based on the EC numbers. CHS: chalcone synthase, [EC:2.3.1.74]; CHI: chalcone isomerase, [EC:5.5.1.6]; FLS: flavonol synthase, [EC:1.14.20.6]; DFR: dihydroflavonol-4-reductase, [EC:1.1.1.219]; LAR: leucoanthocyanidin reductase, [EC:1.17.1.3]; ANR: anthocyanidin reductase, [EC:1.3.1.77]; ANS: anthocyanidin synthase, [EC:1.14.20.4]; F3H: flavanone 3-hydroxylase, [EC:1.14.11.9]; F3’H: flavonoid 3’-hydroxylase, [EC:1.14.14.82]; IFS: isoflavone synthase. **B** A heatmap showing the expression patterns of the enzyme-coding genes in roots, tubers and leaves of *B. striata*. In most cases, there are two or more coding genes. After *z*-score normalization, the heatmap was drawn at a log2 scale from -3 to +3. **C** A heatmap showing the levels of the metabolites in three organs. Based on metabolite quantification, the heatmaps were drawn at a log2 scale from -2 to +2. Flavanone: C00766

In this study, the biosynthetic pathway of flavonoids was referred from the recent report on Ginkgo as mentioned above [12]. A total of six enzymes could find their coding genes from the transcriptome assembly of *B. striata* (colored enzymes, except for the grey ones in Fig. 4A). We noticed that the IFS (isoflavone synthase)-coding gene could not be identified from our transcriptome assembly. According to a recent report, IFSs were specifically existed in the legumes, and the expression of the IFS-coding genes could not be detected in the non-legume plants [23]. Besides, isoflavones, the end products of the IFS-mediated reaction, were legume-specific [24]. Since *B. striata* belongs to the non-legume monocots, failure in detecting the IFS-coding genes should be reasonable. Based on the transcriptome sequencing data, all of the ANR (anthocyanidin reductase, [EC:1.3.1.77])- and CHI (chalcone isomerase, [EC:5.5.1.6])-coding genes were highly expressed in leaves when compared to the other two organs (Fig. 4B and Additional file 2). Besides, according to the metabolic profiling data, flavanones (M225T279 and M225T242), an indispensible precursor for flavonoid synthesis, were highly accumulated in the leaves (Fig. 4C). This result indicated that the flavonoid biosynthetic pathway might be highly active in the leaves of *B. striata*. However, some exceptional cases were observed in this study. For example, all of the genes encoding CHS (chalcone synthase, [EC:2.3.1.74]) and DFR (dihydroflavonol-4-reductase, [EC:1.1.1.219]) were highly active in roots and/or tubers, but not in leaves (Fig. 4B). It was speculated that the precursors for flavonoid biosynthesis might be rich enough to reduce the requirement of the enzyme activities in leaves. Or, the enzymes could be transferred from the underground organs to the leaves as required.

### Terpenoid biosynthetic pathway

**Fig. 5:**
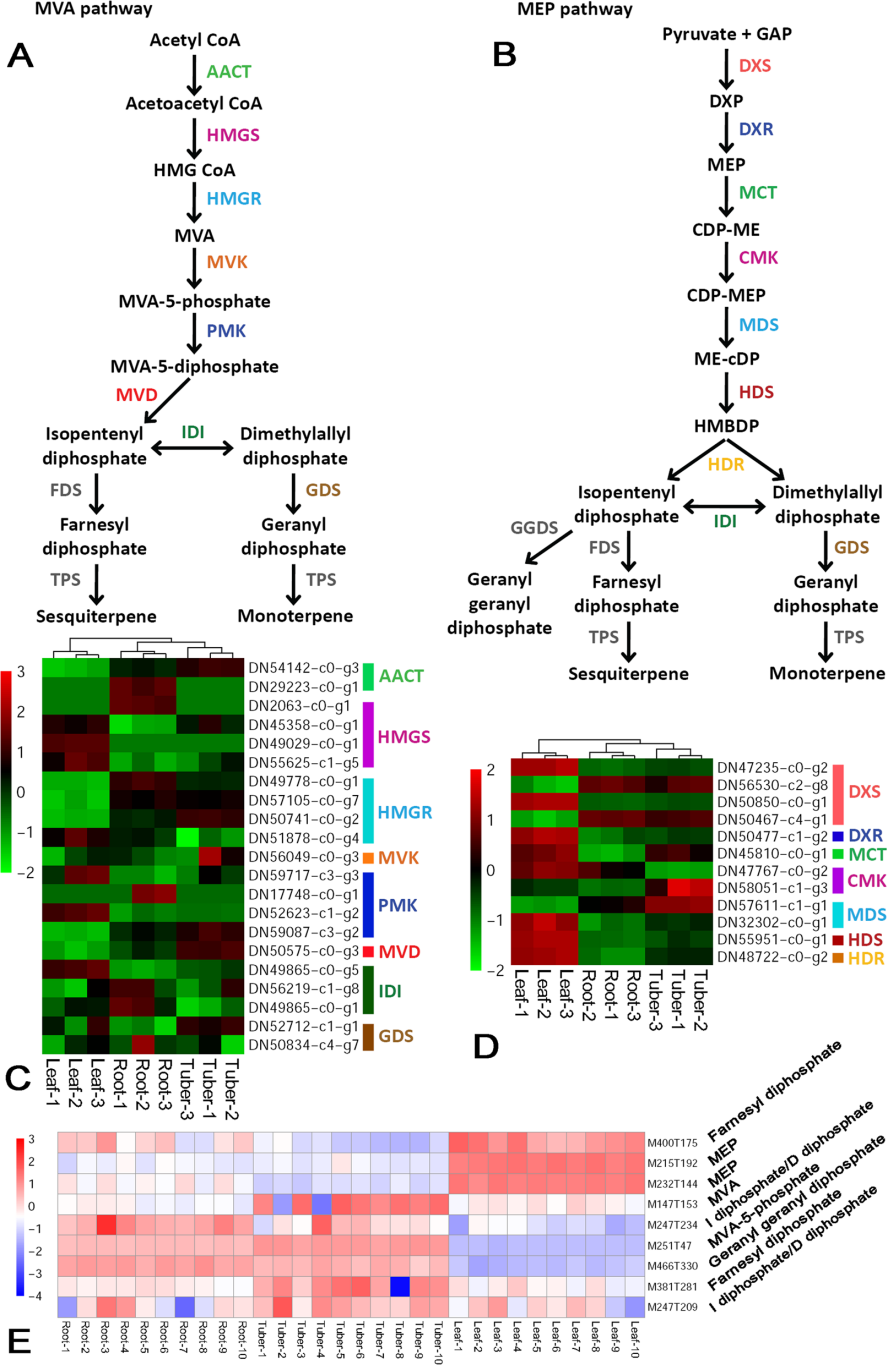
Organ-specific analysis of the levels of the enzyme-coding genes and the metabolites within the biosynthetic pathway of volatile terpenoids in *Bletilla striata*. **A** The previously reported MVA biosynthetic pathway of volatile terpenoids [25]. Based on the transcriptome assembly annotated by the KEGG database, eight enzymes could find their coding genes (colored within the pathway) based on the EC numbers. **B** The previously reported MEP biosynthetic pathway of volatile terpenoids [33]. Based on the transcriptome assembly annotated by the KEGG database, seven enzymes could find their coding genes (colored within the pathway) based on the EC numbers. AACT: acetoacetyl-CoA thiolase, [EC:2.3.1.9]; HMGS: HMG-CoA synthase, [EC:2.3.3.10]; HMGR: HMG-CoA reductase, [EC:1.1.1.34]; MVK: mevalonate kinase, [EC:2.7.1.36]; PMK: phosphomevalonate kinase, [EC:2.7.4.2]; MVD: mevalonate diphosphate decarboxylase, [EC:4.1.1.33]; IDI: isopentenyl diphosphate isomerase, [EC:5.3.3.2]; GDS: geranyl diphosphate synthase, [EC:2.5.1.1]; FDS: farnesyl diphosphate synthase, [EC:2.5.1.10], [EC:2.5.1.68], [EC:2.5.1.92]; TPS: terpene synthase, [EC:4.2.3.47], [EC:4.2.3.49]; DXS: 1-deoxy-D-xylulose-5-phosphate synthase, [EC:2.2.1.7]; DXR: 1-deoxy-D-xylulose-5-phosphate reductoisomerase, [EC:1.1.1.267]; MCT: 2-C-methyl-D-erythritol 4-phosphate cytidylyltransferase, [EC:2.7.7.60]; CMK: CDP-ME kinase, [EC:2.7.1.148]; MDS: 2-C-methyl-D-erythritol 2,4-cyclodiphosphate synthase, [EC:4.6.1.12]; HDS: (E)-4-hydroxy-3-methylbut-2-enyl diphosphate synthase, [EC:1.17.7.1], [EC:1.17.7.3]; HDR: (E)-4-hydroxy-3-methylbut-2-enyl diphosphate reductase, [EC:1.17.7.4]; GGDS: geranyl geranyl diphosphate synthase, [EC:2.5.1.29]. **C** A heatmap showing the expression patterns of the enzyme-coding genes, belonging to the MVA pathway, in roots, tubers and leaves of *B. striata*. In most cases, there are two or more coding genes. After *z*-score normalization, the heatmap was drawn at a log2 scale from -2 to +3. **D** A heatmap showing the expression patterns of the enzyme-coding genes, belonging to the MEP pathway, in three organs of *B. striata*. For some of the enzymes, there are two or more coding genes. After *z*-score normalization, the heatmap was drawn at a log2 scale from -2 to +3. **E** A heatmap showing the levels of the metabolites in three organs. Based on metabolite quantification, the heatmaps were drawn at a log2 scale from -4 to +3. Farnesyl diphosphate: C00448, C16826, C19760; MEP: 2-C-methyl-D-erythritol 4-phosphate, C11434; MVA: mevalonate, C00418; I diphosphate: Isopentenyl diphosphate, C00129, C00235; D diphosphate: Dimethylallyl diphosphate, C00235; MVA-5-phosphate: C01107; Geranyl geranyl diphosphate: C00353

In plants, there are two branches for volatile terpenoid production, including the mevalonate (MVA) and the 2-C-methyl-D-erythritol 4-phosphate (MEP) pathways [25] (Fig. 5A and B). Within the MVA pathway, eight out of ten enzymes could find their coding genes from the transcriptome assembly in this study (colored enzymes, except for the grey ones in Fig. 5A). At first glance, most of the enzyme-coding genes within this pathway were weakly expressed without any obvious organ-specific pattern in *B. striata*. However, it was observed that for AACT (acetoacetyl-CoA thiolase, [EC:2.3.1.9]), HMGS (HMG-CoA synthase, [EC:2.3.3.10]), HMGR (HMGR: HMG-CoA reductase, [EC:1.1.1.34]), PMK (phosphomevalonate kinase, [EC:2.7.4.2]), MVD (MVD: mevalonate diphosphate decarboxylase, [EC:4.1.1.33]), and GDS (GDS: geranyl diphosphate synthase, [EC:2.5.1.1]), at least one coding gene of each enzyme was relatively active in roots and/or tubers (Fig. 5C and Additional file 2). Consistently, the untargeted metabolic profiling data showed that MVA (M147T153) and MVA-5-phosphate (M251T47), both of which were specific to the MVA pathway, were highly accumulated in the roots and/or the tubers (Fig. 5E).

On the other hand, a total of seven enzymes specific to the MEP pathway could find their coding genes from the transcriptome assembly (colored enzymes, except for the grey ones in Fig. 5B). Compared to those genes belonging to the MVA pathway, most of the MEP-specific genes were observed to be highly expressed in the leaves of *B. striata*, such as DXS (1-deoxy-D-xylulose-5-phosphate synthase, [EC:2.2.1.7]), DXR (1-deoxy-D-xylulose-5-phosphate reductoisomerase, [EC:1.1.1.267]), MCT (2-C-methyl-D-erythritol 4-phosphate cytidylyltransferase, [EC:2.7.7.60]), CMK (CDP-ME kinase, [EC:2.7.1.148]), MDS (2-C-methyl-D-erythritol 2,4-cyclodiphosphate synthase, [EC:4.6.1.12]), HDS ((E)-4-hydroxy-3-methylbut-2-enyl diphosphate synthase, [EC:1.17.7.1], [EC:1.17.7.3]), and HDR ((E)-4-hydroxy-3-methylbut-2-enyl diphosphate reductase, [EC:1.17.7.4]) (Fig. 5D and Additional file 2). Accordingly, the metabolic profiling data showed that the pathway-specific metabolites, MEPs (M215T192 and M232T144), exhibited leaf-specific accumulation patterns (Fig. 5E). Additionally, it was noticed that four genes were identified to encode DXS. Among these genes, two genes were highly expressed in leaves, while the other two were relatively active in roots and tubers (Fig. 5D and Additional file 2). A similar case was also observed for the CMK-coding genes. One gene was highly expressed in leaves and roots, while the other one was active in tubers (Fig. 5D and Additional file 2). The complementary organ-specific expression patterns of the above genes indicate that in some cases, the genes encoding the same enzyme may not be functionally redundant. Instead, they may have the organ-specific roles in terpenoid production.

### Indole diterpene alkaloid biosynthetic pathway

**Fig. 6:**
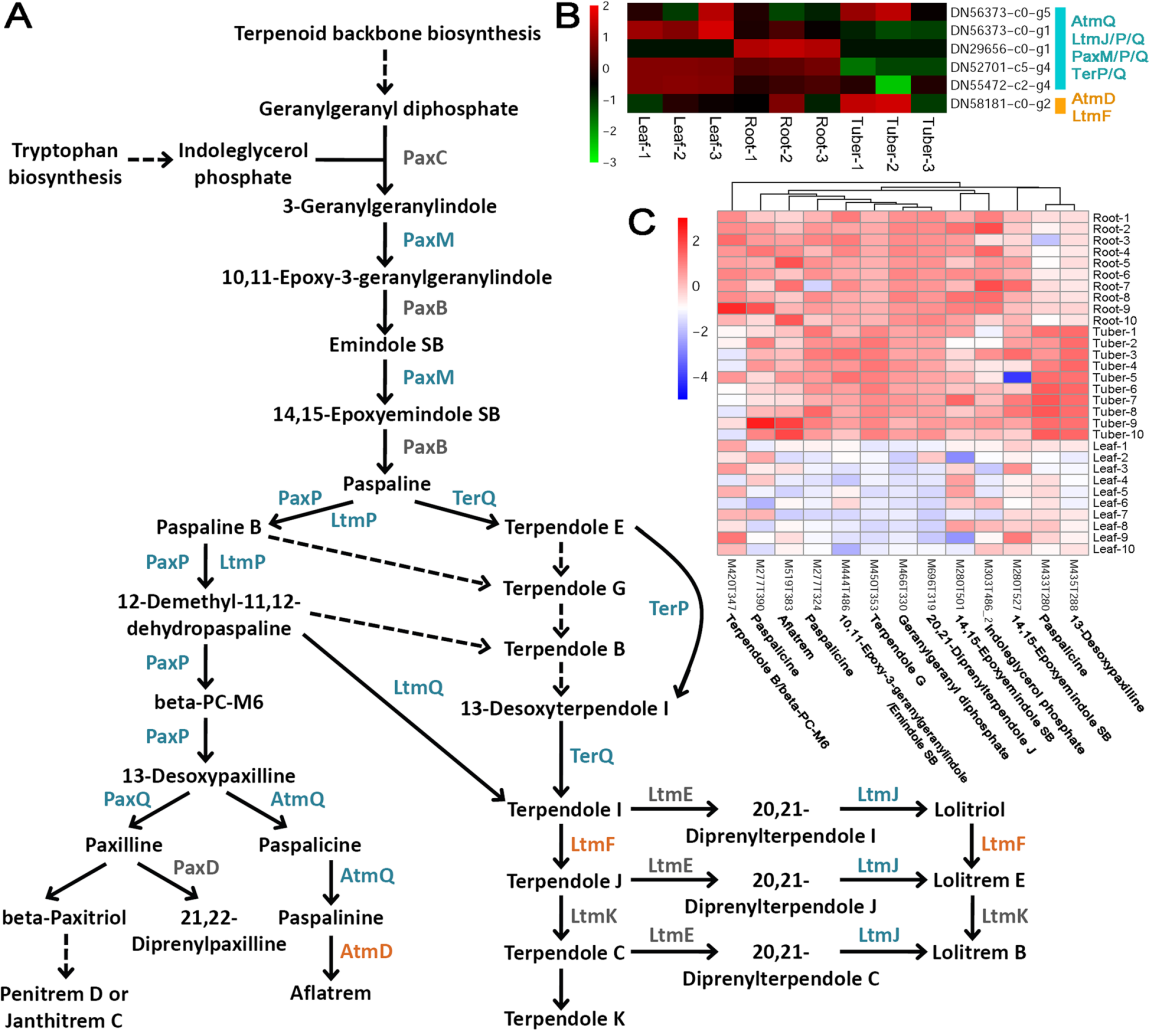
Organ-specific analysis of the levels of the enzyme-coding genes and the metabolites within the biosynthetic pathway of indole diterpene alkaloids in *Bletilla striata*. **A** The biosynthetic pathway of indole diterpene alkaloids referring to the KEGG database (https://www.kegg.jp/kegg-bin/show_pathway?map00403) [13]. Based on the transcriptome assembly annotated by the KEGG database, two types of enzymes could find their coding genes (colored within the pathway) based on the EC numbers. PaxC: geranylgeranyldiphosphate transferase; PaxB: paspaline synthase; PaxD: dimethylallyldiphosphate transferase; LtmE: dimethylallyldiphosphate transferase; PaxM: FAD dependent monooxygenase, [EC:1.14.13.-]; PaxP: cytochrome P450 monooxygenase, [EC:1.14.13.-]; PaxQ: cytochrome P450 monooxygenase, [EC:1.14.13.-]; AtmQ: cytochrome P450 monooxygenase, [EC:1.14.13.-]; TerQ: cytochrome P450 monooxygenase, [EC:1.14.13.-]; TerP: cytochrome P450 monooxygenase, [EC:1.14.13.-]; LtmQ: cytochrome P450 monooxygenase, [EC:1.14.13.-]; LtmP: cytochrome P450 monooxygenase, [EC:1.14.13.-]; LtmJ: cytochrome P450 monooxygenase, [EC:1.14.13.-]; AtmD: dimethylallyldiphosphate transferase, [EC:2.5.1.-]; LtmF: dimethylallyldiphosphate transferase, [EC:2.5.1.-]; LtmK: cytochrome P450 monooxygenase, [EC:1.14.21.-]. **B** A heatmap showing the expression patterns of the enzyme-coding genes in roots, tubers and leaves of *B. striata*. After *z*-score normalization, the heatmap was drawn at a log2 scale from -3 to +2. **C** A heatmap showing the levels of the metabolites in three organs. Based on metabolite quantification, the heatmaps were drawn at a log2 scale from -4 to +2. Terpendole B: C20528; beta-PC-M6: C20535; Paspalicine: C20553; Aflatrem: C20555; 10,11-Epoxy-3-geranylgeranylindole: C20526; Emindole SB: C20527; Terpendole G: C20586; Geranylgeranyl diphosphate: C00353; 20,21-Diprenylterpendole J: C20594; Indoleglycerol phosphate: C03506; 13-Desoxypaxilline: C20531; 14,15-Epoxyemindole SB: C20529

Alkaloids are known to be another important type of the plant secondary metabolites. In this study, we focused on the biosynthesis of the indole diterpene alkaloids. The pathway was referenced from the KEGG database (https://www.kegg.jp/kegg-bin/show_pathway?map00403) [13]. Within this pathway, a total of 11 enzymes could find their potential coding genes from the transcriptome assembly (colored enzymes, except for the grey ones in Fig. 6A). However, according to the KEGG annotations, nine out of the 11 enzymes, including AtmQ (cytochrome P450 monooxygenase), LtmJ/P/Q (cytochrome P450 monooxygenases), PaxM (FAD-dependent monooxygenase), PaxP/Q (cytochrome P450 monooxygenases) and TerP/Q (cytochrome P450 monooxygenases), were totally assigned to the vague ID [EC:1.14.13.-]. And, the remaining two enzymes AtmD and LtmF (both annotated as dimethylallyldiphosphate transferases) were assigned to the vague ID [EC:2.5.1.-]. To solve this issue, these enzymes were classified into two categories, i.e. [EC:1.14.13.-] (cyan in Fig. 6A) and [EC:2.5.1.-] (orange in Fig. 6A). As a result, five genes were identified from the transcriptome assembly to encode the enzymes belonging to the category [EC:1.14.13.-], and one gene was discovered to encode the enzymes belonging to the category [EC:2.5.1.-]. Transcriptome sequencing data showed that the enzyme-coding genes were highly active, and were widely expressed in all of the three organs investigated (Fig. 6B and Additional file 2). However, the metabolic profiling data presented a quite different scene that all of the detected intermediates within the biosynthetic pathway were highly enriched in roots and tubers, but not in leaves (Fig. 6C).

## Discussion

Here, the biosynthetic pathways of BSPs, sterols, flavonoids, terpenoids and alkaloids were comprehensively investigated in different organs of the two-year-old seedlings of *B. striata* through integrated analysis of the transcriptome sequencing data and the untargeted metabolic profiling data. A large portion of the enzyme-coding genes were identified from the transcriptome assembly, allowing us to examine their expression levels in an organ-specific manner. Many intermediates within the metabolic pathways were detected by UPLC-MS/MS, which enabled us to investigate the relationships between enzyme activities and metabolite levels. The results obtained in this study can strengthen the molecular basis for the research on the medicinal value of *B. striata*. However, much more efforts are still needed. For example, according to the KEGG annotations, several key enzymes could only be assigned by vague IDs. In this case, these enzymes could not find their coding genes precisely from the transcriptome assembly, which led to a great obstacle for investigation of the molecular mechanisms underlying secondary metabolite production. In addition to the refinement of enzyme annotations, genome sequencing and annotation should be one of the efficient solutions. There are also some interesting points to be discussed below.

### Omics data-based analysis of the metabolic pathways in *B. striata*: an organ-specific view

In this study, five metabolic pathways were analyzed by investigating the relationships between the expression levels of the enzyme-coding genes and the accumulation levels of the metabolites through an organ-specific way. For some pathways, such as the sterol biosynthetic pathway (Fig. 3B and C) and the MEP pathway for terpenoid synthesis (Fig. 5D and E), the expression patterns of the genes correlate well with the accumulation patterns of the metabolites. In some other cases, the BSP biosynthetic pathway for example, no obvious organ-specific expression pattern was observed for the enzyme-coding genes (Fig. 2B). However, the metabolic profiling data showed that the intermediates belonging to the BSP pathway were highly accumulated in both leaves and tubers (Fig. 2C). For leaves, the enrichment of the BSP-related metabolites could be attributed to their capacity of photosynthesis. But, how about the tubers? Notably, a complete set of the enzyme-coding genes essential for converting fructose to BSPs were identified to be active in the tubers (Fig. 2B), which might partially explain the enrichment of the BSP-related metabolites in this organ.

As previously described in this study, within the sterol biosynthetic pathway, the genes encoding the same enzymes exhibit distinct organ-specific expression patterns (Fig. 5D). For instance, among the four genes encoding the DXS enzyme, two are highly expressed in leaves, while the other two are highly active in roots and tubers. Moreover, one gene encoding CMK is highly expressed in leaves and roots, while the other CMK-coding gene is active in tubers. From this point of view, the enzyme activities could be precisely regulated in different organs possibly at the level of gene transcription.

### Long-range phytochemical transportation inferred from the omics data

In this study, the metabolic data showed that both fructose and sucrose were not only enriched in the photosynthetic organ leaves, but also in the tubers (Fig. 2C). One of the reasonable explanations is that as the primary substrates, sucrose and fructose might be translocated from the source organ leaves to the sink organ tubers for the downstream BSP synthesis. Notably, a complete set of enzyme-coding genes essential for BSP synthesis downstream from fructose were identified to be highly active in the tubers (Fig. 2A). Indeed, it has already been well-studied that long-range transportation of sucrose could be performed from the green tissues to the sites of consumption and storage, such as seeds, tubers and roots of higher plants [26, 27]. Similarly, the long-range transportation from the leaves to the root tips was reported for the isoflavones in soybean (*Glycine max*) [28]. Moreover, intercellular translocation of the plant metabolites and the associated enzymes was reported for the alkaloid biosynthetic pathway [29, 30]. In our study, the CHS-coding genes belonging to the flavonoid biosynthetic pathway were weakly expressed in the leaves of *B. striata* (Fig. 4B). In contrast, the untargeted metabolic profiling data showed that flavanones, the direct downstream product of the CHS-mediated reaction, were highly accumulated in the leaves (Fig. 4C). One possible explanation is that the substrates of the CHS-mediated reaction might be abundant enough to reduce the requirement for the high activity of CHS. Alternatively, CHS might be translocated from the roots and/or the tubers to the leaves as required by the reaction for flavanone production.

## Conclusions

In this study, by integrated analysis of the untargeted metabolic profiling data and the whole-transcriptome sequencing data from roots, tubers and leaves of *B. striata*, the biosynthetic pathways of five different kinds of metabolites were investigated through an organ-specific manner. We hope that this work could strengthen the molecular basis for further studies on breeding and medicinal uses of *B. striata*.

## Methods

### Source of plant

The sample of *B. striata* was collected from Zhangjiajie of Hunan Province, China, in April 2018, and identified by Prof. Aiwen Dong of Jishou University. A voucher specimen (ID-s-201802) was deposited in Dr. Xiao’s Lab, and cultivated in the campus of Hunan Agricultural University. No permission was necessary to collect the above samples.

### Sequencing library construction and data pre-treatment

Leaves, tubers and roots of the two-year-old seedlings of *B. striata* were sampled for total RNA extraction. Three biological replicates were included for each of the three organs. Total RNAs were prepared by using Trizol reagent (Invitrogen, CA, USA) following the manufacturer’s instruction. The total RNA quantity and purity were analyzed by using Bioanalyzer 2100 and RNA 1000 Nano LabChip Kit (Agilent, CA, USA), in order to ensure that the RIN (RNA integrity number) value of each RNA sample was higher than 7.0, concentration was higher than 100 ng/μl, OD260/280 was higher than 1.8, and the total amount of the extracted RNAs were more than 20 μg.

For RNA-seq library construction, poly(A)-tailed transcripts were enriched from 5 μg of total RNAs. Poly-T oligo attached magnetic beads were used for two-round purification. Then, the purified RNAs were fragmented by using divalent cations under elevated temperature. The RNA fragments were reverse-transcribed to generate the final cDNA library according to the protocol of the TruSeq® Stranded mRNA Sample Preparation Kit (Illumina, San Diego, USA). Paired-end sequencing was performed by using Illumina Hiseq4000 (LC Sciences, USA).

Cutadapt (http://code.google.com/p/cutadapt/) and the in-house Perl scripts were used to remove the RNA-seq reads with adaptor contamination or low-quality bases. Then, the read quality was assessed by using FastQC (http://www.bioinformatics.babraham.ac.uk/projects/fastqc/).

### De novo assembly, annotation and heatmap-based gene expression analysis

Trinity [31] was used for *de novo* transcriptome assembly. The RNA-seq data from leaves, tubers and roots of *B. striata* (three biological replicates for each organ) were combinatorially used to assemble the reference transcriptome. The KEGG database [13] was used to annotate the enzyme-coding genes by using the EC number identification. Salmon [32] was used to calculate the expression level (RPKM, reads per kilobase per million) for each transcript [33]. Based the RPKM values, heatmaps were drawn for the enzyme coding genes by using the online tool “Omicstudio” (https://www.omicstudio.cn/) developed by LC Sciences (Hangzhou, China).

### Metabolite extraction for the untargeted metabolomic analysis

Leaves, tubers and roots of the two-year-old seedlings of *B. striata* were sampled for metabolite extraction. For each organ, there are ten biological replicates. The metabolites were extracted with 120 μl of 50% pre-cooled methanol (vortexed for 1 min, incubated at room temperature for 10 min, and then stored at -20 °C overnight). After centrifugation at 4,000 g for 20 min, the supernatants were transferred into the 96-well plates. The samples were stored at -80 °C prior to the UPLC-MS/MS (ultra-performance liquid chromatography—tandem mass spectrometry) analysis. The quality control (QC) sample (six biological replicates) was prepared by mixing an equal volume of each extraction from the three organs.

### UPLC-MS/MS analysis

Firstly, all of the chromatographic separations were performed using the UPLC system (SCIEX, UK). The ACQUITY UPLC T3 column (100 Å, 2.1 mm × 100 mm, 1.8 μm, Waters, UK) was used for the reversed phase separation. The temperature of the column oven was maintained at 35 °C. The flow rate was set to 0.4 ml/min, and the mobile phase consisted of solvent A (water, 0.1% formic acid) and solvent B (acetonitrile, 0.1% formic acid). Gradient elution conditions were set as: 0 to 0.5 min, 5% solvent B; 0.5 to 7 min, 5% to 100% solvent B; 7 to 8 min, 100% solvent B; 8 to 8.1 min, 100% to 5% solvent B; 8.1 to 10 min, 5% solvent B. The injection volume of each sample was 4 μl.

A high-resolution tandem mass spectrometer TripleTOF5600plus (SCIEX, UK) was used to detect metabolites eluted from the ACQUITY UPLC T3 column. The Q-TOF was operated in both positive and negative ion modes. The curtain gas pressure was set as 30 psi, both ion source gas 1 and gas 2 was set as 60 psi, and the interface heater temperature was set as 650 °C. For the positive ion mode, the ionspray voltage floating was set as 5000 V, while for the negative ion mode, it was set as -4500 V. The mass spectrometry data was acquired in information-dependent acquisition (IDA) mode. The TOF mass ranges from 60 to 1,200 Da. The survey scans were acquired in 150 ms. A total of 12 product ion scans would be collected, if a threshold of 100 counts per second (counts/s) with a 1+ charge-state was exceeded. The total cycle time was set as 0.56 second. Four time bins were summed for each scan at a pulser frequency value of 11 kHz through monitoring of the 40 GHz multichannel time-to-digital converter (TDC) detector with four-anode/channel detection. The dynamic exclusion time was set as 4 second. The accuracy of mass acquisition was calibrated for every 20 samples. To ensure the stability of the UPLC-MS/MS analysis, the QC sample as described above was included.

### Bioinformatics analysis of the UPLC-MS/MS data

The software XCMS [34] was used for the pre-treatment of the UPLC-MS/MS data (method: centWave; minfrac: 0.5; snthr: 6; ppm: 30; peakwidth: 5,25; bw2: 5; mzwid: 0.015; mzdiff: 0.01; profStep.OBIWarp: 0.1), including peak picking, peak grouping, retention time correction, second peak grouping, and annotation of isotopes and adducts. The LC−MS raw data files were converted into mzXML format, and then processed by an R package including XCMS, CAMERA [35] and metaX [36]. Each ion was identified based on the retention time (RT)−mass-to-charge ratio (*m/z*) pairs. Peak intensities were recorded, and a three-dimensional matrix containing RT−*m/z* pair-based peak indices, sample names (observations) and ion intensities (variables) was generated.

The online databases KEGG [13], LIPID MAPS [37], and PlantCyc [38] was used to annotate the metabolites by matching the exact *m/z* value of samples with the online resources. If a difference between the measured *m/z* value and the database value was less than 10 parts per million (ppm), the metabolite would be annotated. And the molecular formulas of these metabolites would be identified by isotopic distribution measurement. Additionally, an in-house fragment spectrum library of metabolites was also used to annotate the metabolites. The KEGG C numbers were used to extract the metabolites belonging to the pathways analyzed in this study.

The peak intensities were preprocessed by metaX. The features that were detected in less than 50% of QC samples or 80% of biological samples were discarded. The remaining peaks with missing values were imputed with the *k*-nearest neighbor algorithm in order to improve the data quality. PCA was performed for outlier detection and batch effects evaluation of the pre-processed dataset. Quality control-based robust LOESS signal correction was fitted to the QC data with respect to the order of injection to minimize signal intensity drift over time. The relative standard deviations of the metabolic features were calculated across all of the QC samples, and those > 30% were removed. Based on metabolite quantification, the heatmaps were drawn by using the online tool “Omicstudio” (https://www.omicstudio.cn/) developed by LC Sciences (Hangzhou, China).

## Supplementary Information

**Additional file 1. **Annotations of all assembled genes of *B. striata*.

**Additional file 2. **Expression data of all assembled genes of *B. striata*.

## Data Availability

The transcriptome sequencing data sets of *B. striata* are freely available at NCBI SRA (Sequence Read Archive; https://www.ncbi.nlm.nih.gov/sra/) under the accession ID PRJNA579234. The transcriptome assembly is freely available at http://www.bioinfolab.cn/myj/Assembled%20transcript%20sequences.zip. The untargeted metabolic profiling data sets, both raw and processed data, of *B. striata* are freely available at http://www.bioinfolab.cn/myj/Metabolic%20profiling%20data%20of%20B.%20striata.zip.

## Abbreviations

TCM: Traditional Chinese medicine
RIN: RNA integrity number
KEGG: Kyoto Encyclopedia of Genes and Genomes
RPKM: Reads per kilobase per million
QC: Quality control
IDA: Information-dependent acquisition
TDC: Time-to-digital converter
RT: Retention time
ppm: Parts per million
PCA: Principal component analysis
SRA: Sequence Read Archive
UPLC-MS/MS: Ultra-performance liquid chromatography—tandem mass spectrometry
BSP: *Bletilla striata* polysaccharide
BR: Brassinosteroid
BRX: BREVIS RADIX
scrA: Phosphotransferase system
sacA: Beta-fructofuranosidase
HK: Hexokinase
scrK: Fructokinase
GPI: Glucose-6-phosphate isomerase
pgm: Phosphoglucomutase
UGP2: Uridine-diphosphate glucose pyrophosphorylase
manA: Mannose-6-phosphate isomerase
PMM: Phosphomannomutase
GMPP: Mannose-1-phosphate guanylyltransferase
Suc: Sucrose
Fru: Fructose
Man: Mannose
SQS: Squalene synthase
AltSQE: Alternative squalene epoxidase
LAS: Lanosterol synthase
CAS1: Cycloartenol synthase
SMT1: Sterol 24-C-methyltransferase
SMO1: Plant 4,4-dimethylsterol C-4alpha-methyl-monooxygenase
CPI1: Cycloeucalenol cycloisomerase
CPY51: Sterol 14alpha-demethylase
FK: Delta14-sterol reductase
HYD1: Cholestenol Delta-isomerase
SMT2: 24-methylenesterol C-methyltransferase
SMO2: Plant 4alpha-monomethylsterol monooxygenase
STE1: Delta7-sterol 5-desaturase
DWF5: 7-dehydrocholesterol reductase
DWF1: Delta24-sterol reductase
CYP710A: Sterol 22-desaturase
CHS: Chalcone synthase
CHI: Chalcone isomerase
FLS: Flavonol synthase
DFR: Dihydroflavonol-4-reductase
LAR: Leucoanthocyanidin reductase
ANR: Anthocyanidin reductase
ANS: Anthocyanidin synthase
F3H: Flavanone 3-hydroxylase
F3’H: Flavonoid 3’-hydroxylase
IFS: Isoflavone synthase
AACT: Acetoacetyl-CoA thiolase
HMGS: HMG-CoA synthase
HMGR: HMG-CoA reductase
MVK: Mevalonate kinase
PMK: Phosphomevalonate kinase
MVD: Mevalonate diphosphate decarboxylase
IDI: Isopentenyl diphosphate isomerase
GDS: Geranyl diphosphate synthase
FDS: Farnesyl diphosphate synthase
TPS: Terpene synthase
DXS: 1-deoxy-D-xylulose-5-phosphate synthase
DXR: 1-deoxy-D-xylulose-5-phosphate reductoisomerase
MCT: 2-C-methyl-D-erythritol 4-phosphate cytidylyltransferase
CMK: CDP-ME kinase
MDS: 2-C-methyl-D-erythritol 2,4-cyclodiphosphate synthase
HDS: (E)-4-hydroxy-3-methylbut-2-enyl diphosphate synthase
HDR: (E)-4-hydroxy-3-methylbut-2-enyl diphosphate reductase
GGDS: Geranyl geranyl diphosphate synthase
MVA: Mevalonate
I diphosphate: Isopentenyl diphosphate
D diphosphate: Dimethylallyl diphosphate
MEP: 2-C-methyl-D-erythritol 4-phosphate
PaxC: Geranylgeranyldiphosphate transferase
PaxB: Paspaline synthase
PaxD: Dimethylallyldiphosphate transferase
LtmE: Dimethylallyldiphosphate transferase
PaxM: FAD dependent monooxygenase
PaxP: Cytochrome P450 monooxygenase
PaxQ: Cytochrome P450 monooxygenase
AtmQ: Cytochrome P450 monooxygenase
TerQ: Cytochrome P450 monooxygenase
TerP: Cytochrome P450 monooxygenase
LtmQ: Cytochrome P450 monooxygenase
LtmP: Cytochrome P450 monooxygenase
LtmJ: Cytochrome P450 monooxygenase
AtmD: Dimethylallyldiphosphate transferase
LtmF: Dimethylallyldiphosphate transferase
LtmK: Cytochrome P450 monooxygenase
